# Supplementary material for: AUTOENCODIX: a generalized and versatile framework to train and evaluate autoencoders for biological representation learning and beyond
Source: Nat Comput Sci. 2025 Dec 9;6(1):96–108. doi: 10.1038/s43588-025-00916-4 (PMC12855006; doi:10.1038/s43588-025-00916-4)
Supplement: Supplementary file 1 — Supplementary Information Supplementary Tables 1–6, Figs. 1–6 and text. [file 43588_2025_916_MOESM1_ESM.pdf]

nature computational science

Supplementary information https://doi.org/10.1038/s43588-025-00916-4

AUTOENCODIX: a generalized and versatile framework to train and evaluate autoencoders for biological representation learning and beyond

In the format provided by the authors and unedited

SUPPLEMENTARY INFORMATION

Data Sets

Data set name	Reference	Data modality	# samples	# features	pre-filtering	subtypes
TCGA		RNASeq	10059		no	32 cancer subtypes
		Mutational score	9866		no	
		DNA Methylation	10729		no	
		combined	9267			
sc-Cortex		sc-RNASeq	24437	6000	yes, via scanpy	15 cell types
		sc-ATACSeq	45549	6000	yes, via scanpy	
		combined	24437			
Celegans		Proteome	260	279	no	260 time points
		Image	260	128x128 pixel	no	260 time points
MNIST		Image	3230	64 x 64 pixel	no	0-4 digits

Supplementary Table 1: Overview of data sets used in experiments.

Overview experimental set-up and hyperparameterization

Experiment	Data Set	Data modalities	Number of Features	Feature scaling	Feature filtering	Learning Rate	Epochs	Batch size	Beta value	Drop-out	Annealing	Latent dimensions	Gamma	Delta paired	Delta class
Exp1 - beta influence	TCGA	all three modalities	2000 each DM	Standard scaler	variance-based	0.0001	1000	256	0, 0.01, 0.1, 1, or 10	0.1	logistic	2	-	-	-
Exp2 - architectures	TCGA	RNASeq	6000	Standard scaler	variance-based	0.0001	1000	256	1 or 0.1 or 0.01	0.1	logistic	2 or 8 or 29	-	-	-
		Mutational score	6000	Standard scaler	variance-based	0.0001	1000	256	1 or 0.1 or 0.01	0.1	logistic	2 or 8 or 29	-	-	-
		DNA methylation	6000	Standard scaler	variance-based	0.0001	1000	256	1 or 0.1 or 0.01	0.1	logistic	2 or 8 or 29	-	-	-
		RNA + MUT	3000 each DM	Standard scaler	variance-based	0.0001	1000	256	1 or 0.1 or 0.01	0.1	logistic	2 or 8 or 29	-	-	-
		RNA + METH	3000 each DM	Standard scaler	variance-based	0.0001	1000	256	1 or 0.1 or 0.01	0.1	logistic	2 or 8 or 29	-	-	-
		METH + MUT	3000 each DM	Standard scaler	variance-based	0.0001	1000	256	1 or 0.1 or 0.01	0.1	logistic	2 or 8 or 29	-	-	-
		RNA+METH+MUT	1500 each DM	Standard scaler	variance-based	0.0001	1000	256	1 or 0.1 or 0.01	0.1	logistic	2 or 8 or 29	-	-	-
											logistic		-	-	-
	sc-Cortex	sc-RNASeq	3000	Standard scaler	variance-based	0.0001	1000	256	1 or 0.1 or 0.01	0.1	logistic	2 or 8 or 29	-	-	-
		sc-ATACSeq	3000	Standard scaler	variance-based	0.0001	1000	256	1 or 0.1 or 0.01	0.1	logistic	2 or 8 or 29	-	-	-
		RNA+METH	1500 each DM	Standard scaler	variance-based	0.0001	1000	256	1 or 0.1 or 0.01	0.1	logistic	2 or 8 or 29	-	-	-
Exp3 - Ontix robustness	TCGA	RNA+METH+MUT	1500 each DM	Standard scaler	variance-based	[0.01 - 1e-6]	1000	256	[0.001 - 1.0]	[0.0 - 0.9]	logistic	29 Reactome or 25 Chromosomes	-	-	-
	sc-Cortex	RNA+METH	1500 each DM	Standard scaler	variance-based	[0.01 - 1e-6]	1000	256	[0.001 - 1.0]	[0.0 - 0.9]	logistic	29 Reactome or 25 Chromosomes	-	-	-
Exp4 - Celegans	Celegans	TF proteom + Img	279 TF; 128x128 pixels	MinMax scaler	no filtering	0.0005	1000	32	0.01	0.1	logistic	32	1.25	0.7	0.0
Exp5 - TCGA-MNIST	TCGA-MNIST	RNA + MNIST	4000 genes; 64x64 pixels	MinMax scaler	variance-based	0.0005	1000	512	0.2	0.5	logistic	12	10	0	10
Exp6-1 - TCGA METH RNA	TCGA	RNA + METH	4000 each DM	MinMax scaler	variance-based	0.0005	1000	512	0.1	0.2	logistic	12	10	10	0
Exp6-2 - TCGA varix	TCGA	RNA	4000 each DM	MinMax scaler	variance-based	0.0005	1000	512	0.1	0.2	logistic	12	-	-	-

Supplementary Table 2: Overview of experiments and hyperparameter configuration.

Details architecture comparison

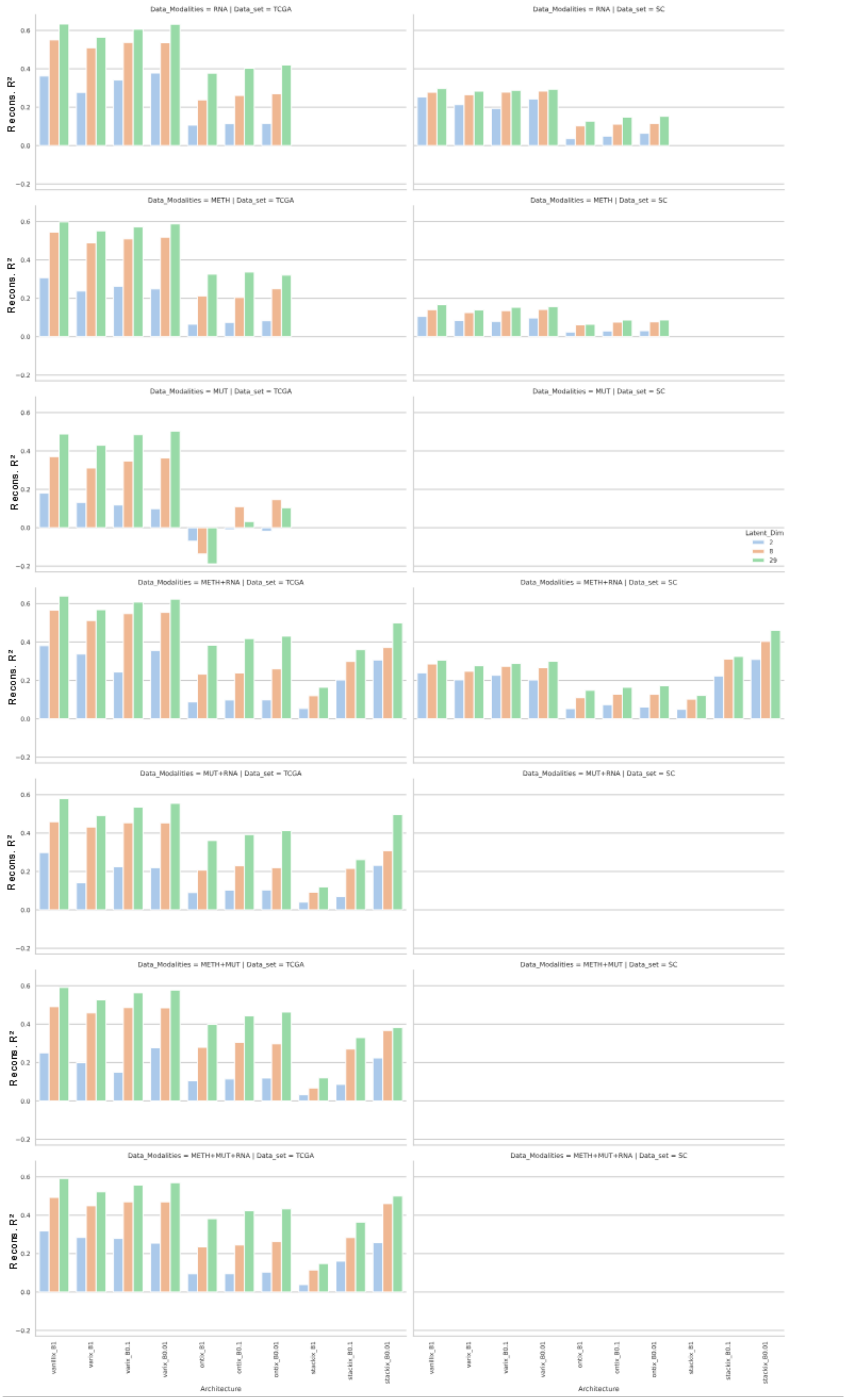

Supplementary Figure 1: Detailed reconstruction capability of AE types

**TCGA** variables	**sc-Cortex** variables
$CANCER\_TYPE\_ACRONYM$	$author\_cell\_type$
$TMB\_NONSYNONYMOUS$	age_group
AGE	sex
SEX	
$AJCC\_PATHOLOGIC\_TUMOR\_STAGE\_SHORT$	
OS_STATUS	
OS_MONTHS	
DFS_STATUS	
PFS_STATUS	
MSI_SCORE_MANTIS	
$ANEUPLOIDY\_SCORE$	

Supplementary Table 3: Overview of tasks (annotation labels) for supervised learning on embeddings.

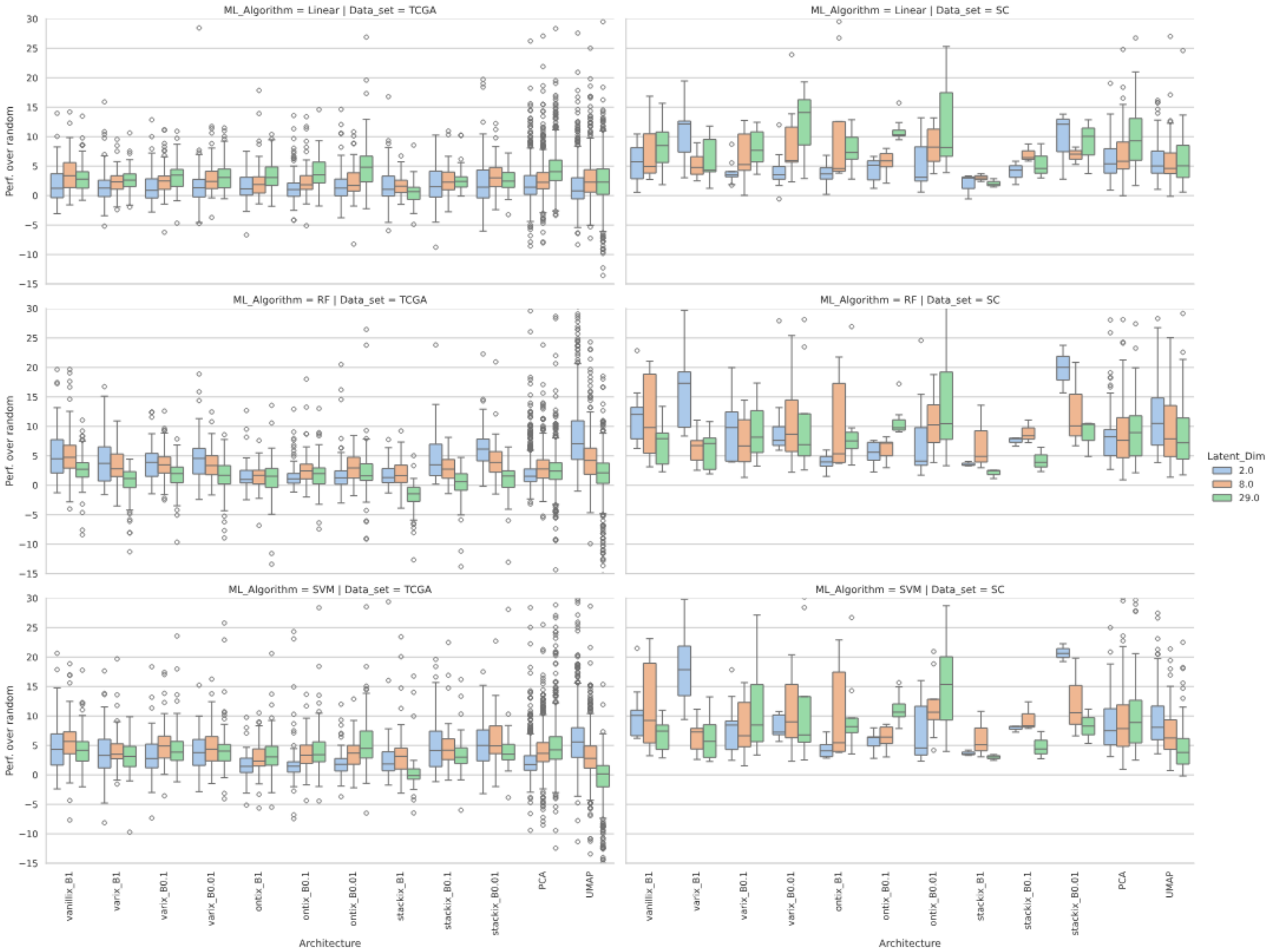

Supplementary Figure 2: Detailed embedding performance of AE types. Box plots show the median, interquartile range, whiskers extending to 1.5× the interquartile range, and outliers as points.

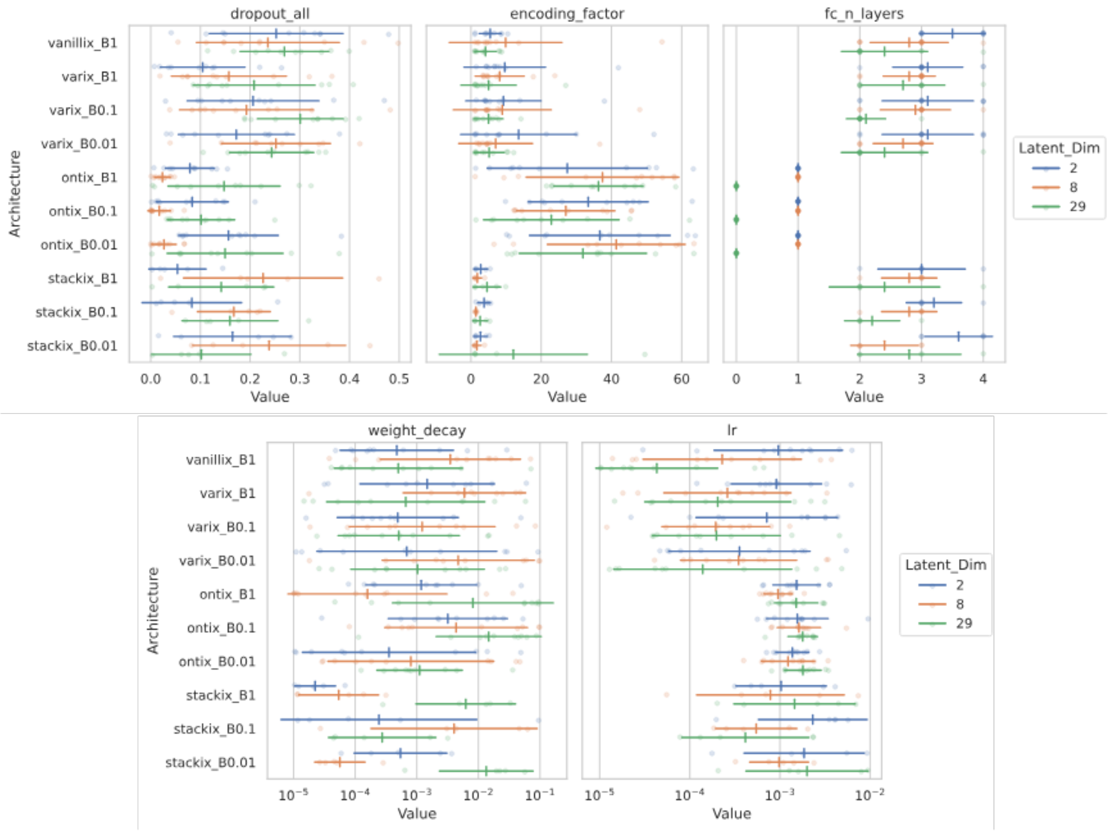

Supplementary Figure 3: Value range of optimized hyperparameters after tuning. Vertical bold line represents mean value and horizontal bold line the standard deviation.

Ontology-based VAE on single-cell data

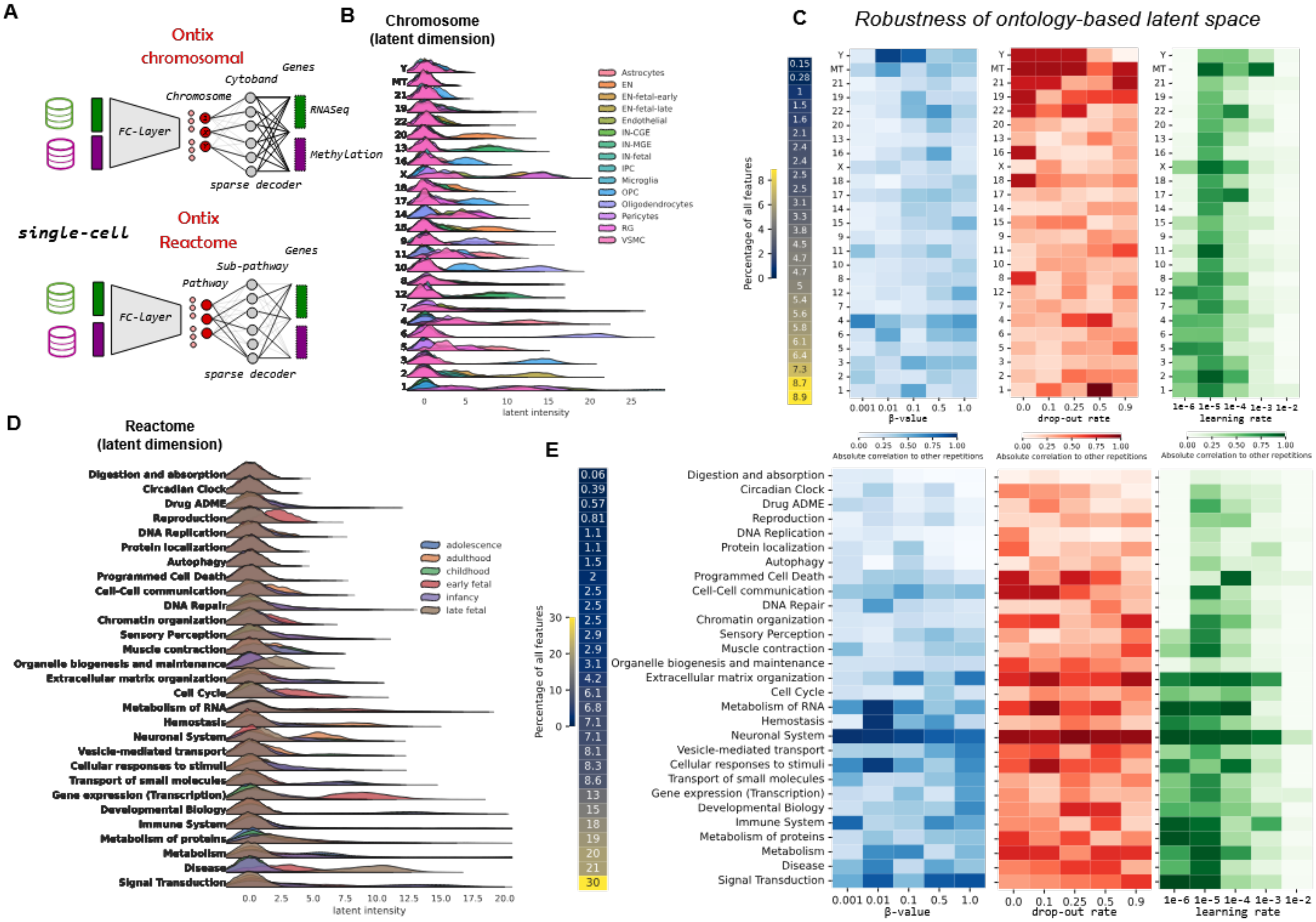

Supplementary Figure 4: Ontix: explainability and robustness of biologically-informed decoder. **(A)** Testing two types of ontologies on the sc-Cortex dataset: chromosomal-based and Reactome-pathway-based latent dimensions. **(B)** and **(D)** ridge-line plots of latent intensity distributions, based on selected examples of sample classes (cell type and development stage). **(C)** and **(E)** robustness of embeddings in relation to hyperparameters. Robustness: mean absolute Pearson correlation between five independent training runs with a randomized data split and weight initialization.

Cross-modal VAE evaluation details

To quantify the translation capabilities of the cross-modal autoencoder, we implemented two evaluation experiments. Firstly, we compared the reconstructions of three different cross-modal autoencoders with the original images by calculating the pixel-wise MSE. The three architectures were: (a) the cross-modal autoencoder as explained in the Material and Methods sections, (b) a reference Image VAE without translation and additional loss terms for latent space alignment, and (c) the cross-modal autoencoder without translation using image encoding and decoding. Figure 5**A** shows the aggregated MSEs over all time points and Figure 5 **B** shows the MSE for each time point reconstruction. Interestingly, MSE on test images is lower for translated images and direct image reconstruction of the cross-modal autoencoder in comparison to a pure Image VAE.

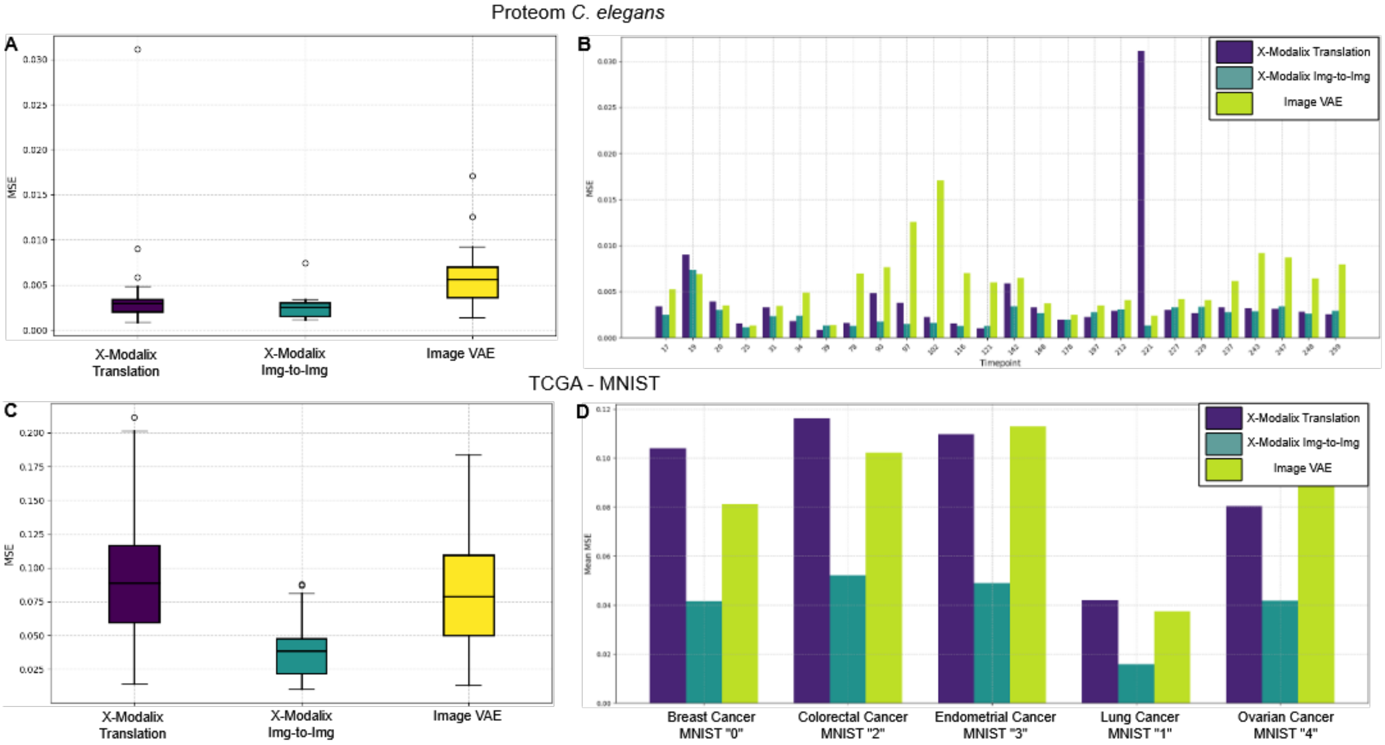

Supplementary Figure 5: Quantitative evaluation of the cross-modal autoencoder. **(A)** MSE of all predictions of the test samples compared with the original images for different autoencoder scenarios depicted as boxplot for the *C. elegans* experiment. i**(B)** MSE between original and reconstructed test samples at each time point for three different autoencoder scenarios for *C. elegans* experiment. **(C)** As figure A, but for the TCGA/MNIST example **(D)** As figure B, but for the TCGA/MNIST example.

Secondly, we trained a basic convolutional neural network (CNN) regression model on the original training images to predict the time point of each image. The rationale behind this approach was that if the translation performs effectively, the trained model should achieve similar performance when inferring time points from both the original test data and the reconstructed test data. Model performance was evaluated using the R^2 metric, as reported in Table 4. Additionally, we also trained the CNN with the reconstructed train data and evaluated it with the reconstructed test data, to investigate whether the differences in performance come from a distribution shift, or from changed task difficulty (shown in the row Test Reconstructed (trained_recons))

**Dataset**	$\mathbf{R^2}$
Train Original	0.9485
Train Reconstructed	-0.9275
Validation Original	0.9249
Validation Reconstructed	-1.6334
Test Original	0.9098
Test Reconstructed	-0.7454
Test Reconstructed (trained_recons)	0.8961

Supplementary Table 4: Model evaluation R2 metric across datasets

We use a pre-trained VGG16 model from torchvision [1] with default weights as our CNN. We froze all layers except the last layer to allow transfer learning and retrained the model for 50 epochs.

The results presented in Table 4 offer a clear comparison of the model's performance on the original and reconstructed datasets. For both the training and test sets, the R^2 values for the original images were consistently high ($R^2 > 0.9$), indicating that the model effectively learned to predict the time points from the original images. However, the performance on the reconstructed data was sig-

[1] https://docs.pytorch.org/vision/main/models/generated/torchvision.models.vgg16.html

nificantly poorer, with negative R^2 values. This disparity suggests that the reconstructed images originate from a different distribution than the original images, leading to the model's inability to generalize effectively. When considered alongside the results depicted in Figures 5 **A** and **B**, we conclude that while the cross-modal translation method performs comparably to traditional reconstruction techniques, it still alters the data distribution in a way that impairs the ability of AI models to learn accurately. Interestingly, while CNN do not generalize, reconstructed images for human eyes are highly similar to original images with no detectable distribution shift.

We performed the same experiments for the TCGA/MNIST example as shown in Figures 5 **C** and **D** and Table 5. The only difference is that we adjusted the VGG16 net to be a classifier instead of a regression model. As assessed by the F1 score (see Table 5), the model's performance was significantly better on the original datasets than the reconstructed ones.

Table 5 shows that the classifier achieved an F1 score of 0.99 on the original training data and 0.97 on the original test data, indicating high classification accuracy. However, on the reconstructed datasets, the F1 score dropped to 0.68 for the training set and 0.73 for the test set, showing a similar trend to the regression experiments, but not as stark. This is likely because classifying digits is easier than inferring time points.

**Dataset**	**F1 Score**
Train Original	0.9903
Train Reconstructed	0.6843
Validation Original	0.9795
Validation Reconstructed	0.6926
Test Original	0.9718
Test Reconstructed	0.7280
Test Reconstructed Trained on Reconstructed	0.5404

Supplementary Table 5: F1 Score of models on various datasets

A noteworthy observation from both experiments is that even when the model was trained on the reconstructed images, the regression and classification performance still decreased compared to the original data. Since reconstructed images are visually very similar to original images for human eyes, CNNs are very likely picking up other fine-granular patterns and pixel distributions which do not represent the core of cellular structures or digits in the images. Hence, they fail to generalize on reconstructed images.

Class Frequencies: The datasets contain different numbers of cases across five cancer types. Table 6 shows the class frequencies per split.

**Cancer Type**	**Training Set**	**Validation Set**	**Test Set**
Non-Small Cell Lung Cancer	707	190	94
Breast Cancer	715	231	119
Endometrial Cancer	417	109	58
Colorectal Cancer	422	115	52
Ovarian Epithelial Tumor	209	61	30

Supplementary Table 6: Class frequencies across cancer types in training, validation, and test sets

Cross-modal VAE on TCGA data combined with MNIST digits

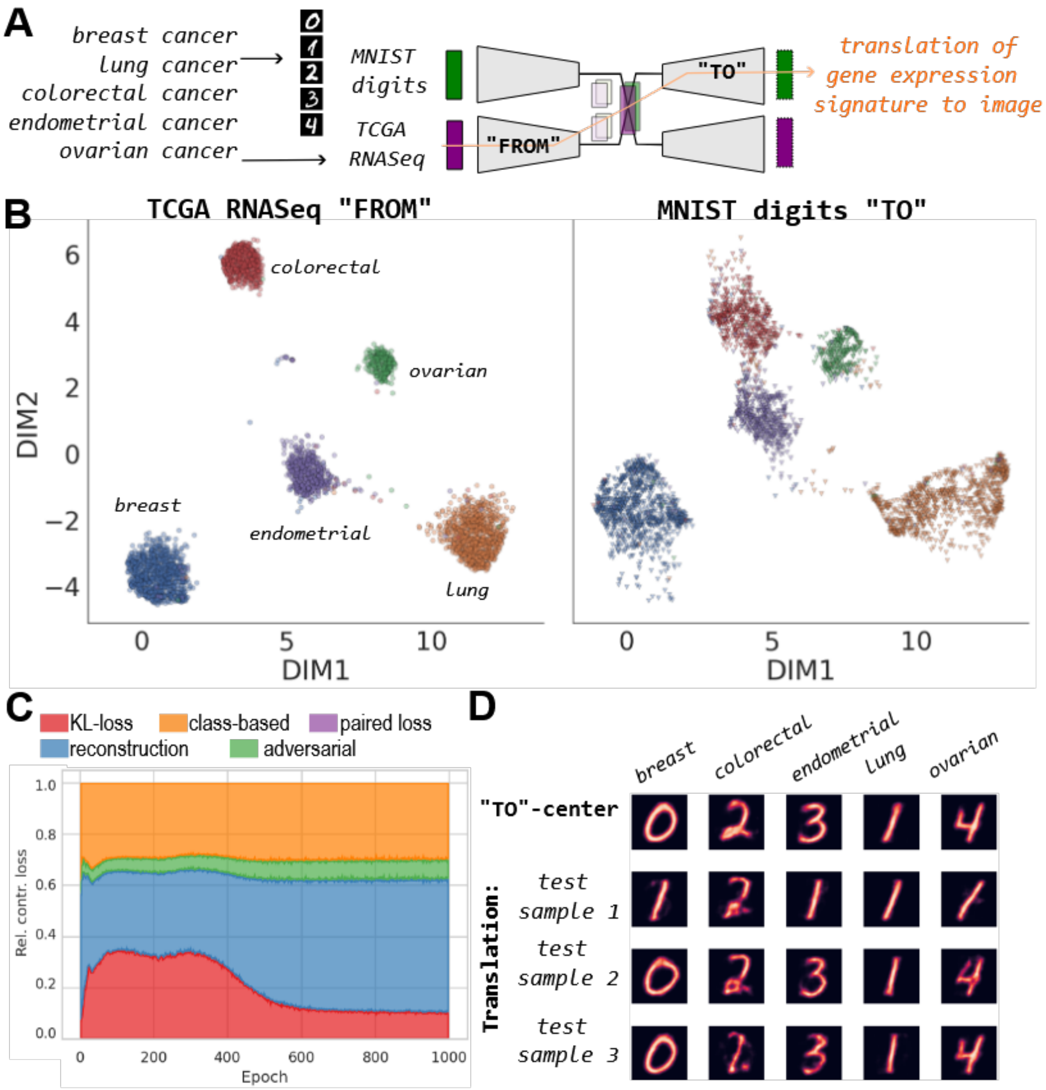

Supplementary Figure 6: X-Modalix on TCGA and MNIST. **A** Cross-modal VAE of TCGA RNASeq data of selected cancer types combined and randomly assigned to MNIST digits for each class. In **B** aligned latent spaces as 2D UMAP representation and in **C** the relative contribution of each loss terms (without annealing factor) during training over 1000 epochs calculated on samples which are not used for training. Comparison translated images from RNASeq signatures in **D** for class centers and random test samples from TCGA RNASeq translated to images of digits.
