# Supplementary material for: AUTOENCODIX: a generalized and versatile framework to train and evaluate autoencoders for biological representation learning and beyond
Source: Nat Comput Sci. 2025 Dec 9;6(1):96–108. doi: 10.1038/s43588-025-00916-4 (PMC12855006; doi:10.1038/s43588-025-00916-4)
Supplement: Supplementary file 2 — Reporting Summary [file 43588_2025_916_MOESM2_ESM.pdf]

nature portfolio

Corresponding author(s): Jan Ewald

Last updated by author(s): Sep 24, 2025

Reporting Summary

Nature Portfolio wishes to improve the reproducibility of the work that we publish. This form provides structure for consistency and transparency in reporting. For further information on Nature Portfolio policies, see our Editorial Policies and the Editorial Policy Checklist.

Statistics

For all statistical analyses, confirm that the following items are present in the figure legend, table legend, main text, or Methods section.

n/a	Confirmed	
☐	☒	The exact sample size (*n*) for each experimental group/condition, given as a discrete number and unit of measurement
☐	☒	A statement on whether measurements were taken from distinct samples or whether the same sample was measured repeatedly
☐	☒	The statistical test(s) used AND whether they are one- or two-sided<br>*Only common tests should be described solely by name; describe more complex techniques in the Methods section.*
☐	☒	A description of all covariates tested
☐	☒	A description of any assumptions or corrections, such as tests of normality and adjustment for multiple comparisons
☐	☒	A full description of the statistical parameters including central tendency (e.g. means) or other basic estimates (e.g. regression coefficient) AND variation (e.g. standard deviation) or associated estimates of uncertainty (e.g. confidence intervals)
☐	☒	For null hypothesis testing, the test statistic (e.g. *F*, *t*, *r*) with confidence intervals, effect sizes, degrees of freedom and *P* value noted<br>*Give P values as exact values whenever suitable.*
☐	☒	For Bayesian analysis, information on the choice of priors and Markov chain Monte Carlo settings
☐	☒	For hierarchical and complex designs, identification of the appropriate level for tests and full reporting of outcomes
☐	☒	Estimates of effect sizes (e.g. Cohen's *d*, Pearson's *r*), indicating how they were calculated

Our web collection on statistics for biologists contains articles on many of the points above.

Software and code

Policy information about availability of computer code

Data collection	We provide a reproducibility code repository with all relevant scripts under: https://github.com/jan-forest/autoencodix-reproducibility The collected data from sources which are specified in the main text is stored via Zenodo under: https://zenodo.org/records/13691753
Data analysis	All computational experiments and analysis including plot generation can be reproduced using the code under our reproducibility repository: https://github.com/jan-forest/autoencodix-reproducibility

For manuscripts utilizing custom algorithms or software that are central to the research but not yet described in published literature, software must be made available to editors and reviewers. We strongly encourage code deposition in a community repository (e.g. GitHub). See the Nature Portfolio guidelines for submitting code & software for further information.

Data

Policy information about availability of data

All manuscripts must include a data availability statement. This statement should provide the following information, where applicable:

- Accession codes, unique identifiers, or web links for publicly available datasets
- A description of any restrictions on data availability
- For clinical datasets or third party data, please ensure that the statement adheres to our policy

No data have been generated for this research. Publicly available datasets have been used for framework development and analysis. The collected and pre-formatted data,

as described under the section Datasets, is stored and hosted via Zenodo https://zenodo.org/records/15518831. Source data are provided with this paper.

Human research participants

Policy information about studies involving human research participants and Sex and Gender in Research.

Reporting on sex and gender	Does not apply. Only public available data sets have been used and no data have been generated via own studies.
Population characteristics	Does not apply. Only public available data sets have been used and no data have been generated via own studies.
Recruitment	Does not apply. Only public available data sets have been used and no data have been generated via own studies.
Ethics oversight	Does not apply. Only public available data sets have been used and no data have been generated via own studies.

Note that full information on the approval of the study protocol must also be provided in the manuscript.

Field-specific reporting

Please select the one below that is the best fit for your research. If you are not sure, read the appropriate sections before making your selection.

☒ Life sciences ☐ Behavioural & social sciences ☐ Ecological, evolutionary & environmental sciences

For a reference copy of the document with all sections, see nature.com/documents/nr-reporting-summary-flat.pdf

Life sciences study design

All studies must disclose on these points even when the disclosure is negative.

Sample size	Does not apply. Only public available data sets have been used and no data have been generated via own studies.
Data exclusions	Does not apply. Only public available data sets have been used and no data have been generated via own studies.
Replication	Does not apply. Only public available data sets have been used and no data have been generated via own studies.
Randomization	Does not apply. Only public available data sets have been used and no data have been generated via own studies.
Blinding	Does not apply. Only public available data sets have been used and no data have been generated via own studies.

Reporting for specific materials, systems and methods

We require information from authors about some types of materials, experimental systems and methods used in many studies. Here, indicate whether each material, system or method listed is relevant to your study. If you are not sure if a list item applies to your research, read the appropriate section before selecting a response.

Materials & experimental systems

n/a	Involved in the study	
☒	☐	Antibodies
☒	☐	Eukaryotic cell lines
☒	☐	Palaeontology and archaeology
☒	☐	Animals and other organisms
☒	☐	Clinical data
☒	☐	Dual use research of concern

Methods

n/a	Involved in the study	
☒	☐	ChIP-seq
☒	☐	Flow cytometry
☒	☐	MRI-based neuroimaging
